# Evaluation of Antibiotic Susceptibility of Gram-Positive Anaerobic Cocci Isolated from Cancer Patients of the N. N. Blokhin Russian Cancer Research Center

**DOI:** 10.1155/2015/648134

**Published:** 2015-12-21

**Authors:** Irina I. Shilnikova, Natalia V. Dmitrieva

**Affiliations:** Department of Health, N. N. Blokhin Russian Cancer Research Center, 24 Kashirskoe Shosse, Moscow 115478, Russia

## Abstract

In total, 81 nonduplicate gram-positive anaerobic cocci (GPAC) were involved in this study. The GPAC were isolated from samples collected from cancer patients between 2004 and 2014. Species identification was carried out by matrix-assisted laser desorption ionization time of flight mass spectrometry (MALDI-TOF MS). The majority of isolates were identified as* Finegoldia magna* (47%) and* Peptoniphilus harei* (28%). The susceptibility of six species of GPAC was determined for eight antibiotics according to *E*-test methodology. Furthermore, all isolates were susceptible to imipenem, vancomycin, and linezolid. Susceptibility to penicillin G, amoxicillin/clavulanate, metronidazole, ciprofloxacin, and levofloxacin varied for different species. One* Finegoldia magna* isolate was multidrug-resistant (i.e., parallel resistance to five antimicrobial agents, including metronidazole, was observed). Two* Parvimonas micra* isolates were highly resistant to metronidazole (MIC 256 *μ*g/mL) but were sensitive to other tested antibiotics.

## 1. Introduction

Gram-positive anaerobic cocci (GPAC) account for approximately 25–30% of all isolated anaerobic bacteria from clinical specimens [1]. GPAC are the part of the commensal flora, but they often can become opportunistic pathogens. GPAC can be isolated from a wide variety of sites such as blood, abscesses, and infections of skin and soft tissue, joints and bones, mouth, respiratory tract, gut, and urogenital tract.

The taxonomy of this heterogeneous group has undergone extensive changes in the last decades due to the development and application of molecular identification methods, including PCR and the sequencing of the 16S rRNA gene [2, 3]. A new method has been recently introduced for bacterial identification, matrix-assisted laser desorption ionization time of flight mass spectrometry (MALDI-TOF MS). This rapid, precise, and cost-effective technology has been successfully implemented for accurately identifying GPAC [4].

Since 1998, the genus* Peptostreptococcus* has been reclassified into several new genera and species. At present, the important genera of GPAC that are commonly isolated from clinical material are* Peptostreptococcus* (mainly* Pe. anaerobius*),* Peptoniphilus* (*Pt. harei*),* Anaerococcus* (*A. vaginalis*),* Finegoldia magna*, and* Parvimonas micra* [5, 6]. They are frequently isolated from both local and systemic infections. GPAC are mostly isolated from polymicrobial infections. However, they can also be isolated in a pure culture, especially* F. magna*.

GPAC are mainly susceptible to *β*-lactam/*β*-lactamase inhibitors, carbapenems, and chloramphenicol. Nonetheless, antibiotic susceptibility can vary in different species [7]. GPAC have variable resistance to penicillin (7–10%), clindamycin (7–20%), and metronidazole (5–10%). They are also resistant to tetracycline and erythromycin. Owing to the differences in antibiotic susceptibility among GPAC species, it is very important to correctly identify isolates and test their susceptibility in order to administer effective antibacterial therapy.

The aims of this study were to determine the species diversity of GPAC isolated from cancer patients with infectious complications, analyse the sensitivity of these anaerobes to antibiotics, and calculate the resistance levels in our hospital.

## 2. Materials and Methods

### 2.1. Specimen Collection and Growth Conditions

The study was performed at the 1,500-bed N. N. Blokhin Cancer Research Center, which treats adults and children in Moscow, Russia, under auspices of the Department of Health. All clinical specimens included in this study were collected between June 2004 and October 2014 from patients with various malignancies. The clinical samples were transported within two hours of collection to a laboratory without the use of special anaerobic transport systems. They were inoculated onto Schaedler Agar (bioMerieux, France), supplemented with hemin, menadione, and 5% blood. Furthermore, they were placed in a thioglycollate broth (bioMerieux, France) for enrichment. All plates were incubated anaerobically using the jar and AnaeroGen systems (Oxoid, UK) at 37°C for 48 hours. Each colony morphotype was simultaneously subcultured on Schaedler Agar plates and blood agar plates. The primary plates were reincubated anaerobically while the subcultures were incubated aerobically to detect any bacteria that were not strictly anaerobic. Identification of strains isolated until 2012 (when it was still impossible to use the MALDI-TOF MS device) was done by using gram staining and the Rapid ID 32A (bioMerieux, France), VITEK 2 (bioMerieux, France), or MicroScan WalkAway (Siemens, UK) systems. These strains were then rechecked through the MALDI-TOF MS. The GPAC strains were stored in 20% skim milk at −70°C until their use in this study.

### 2.2. MALDI-TOF Identification of Isolates

In order to ensure final identification of isolates at the species level, the matrix-assisted laser desorption ionization time of flight mass spectrometry (MALDI-TOF MS) was employed. Pure cultures of each frozen strain were subcultured twice on a blood agar anaerobically and analysed based on the direct transfer method. Single or few colonies were smeared directly onto a metallic target plate in a thin film using an inoculation needle. All spots were overlaid with 1 *μ*L of an *α*-cyano-4-hydroxycinnamic acid matrix solution in an organic solvent (50% acetonitrile and 2,5% trifluoroacetic acid). Each strain was applied in three spots. Then, the target plate was left to dry at room temperature.* Escherichia coli* ribosomal proteins (bacterial test standard, Bruker Daltonik, Germany) were used as a positive control and calibration standard. Mass spectra were obtained using MicroFlex LT mass spectrometer (Bruker Daltonik, Germany) and then analysed with Biotyper 3.0 software within the FlexControl program. For bacterial identification, the peak list for an unknown isolate is compared with the reference library of spectra. A library of 5,629 standard spectra (version 4.0.0.1) was used. The MALDI-TOF Biotyper output is a log (score) in the range of 0 to 3.0. According to the criteria proposed by the manufacturer, a log (score) between 1.7 and 1.99 indicates genus level identification, while a log (score) ≥2 indicates species level identification.

### 2.3. Antimicrobial Susceptibility Testing

Minimal inhibitory concentration (MIC) was determined by using the M.I.C. Evaluator (M.I.C.E.), a new gradient endpoint device (Oxoid, England). The M.I.C.E. strip operates on a principle similar to that of the original *E*-test device [8]. Eight antimicrobial agents were tested: metronidazole, amoxicillin/clavulanate, imipenem, penicillin G, vancomycin, linezolid, ciprofloxacin, and levofloxacin. The inoculum for each isolate was prepared in a saline solution at a density equivalent to the 1.0 McFarland Standard. After the inoculation of the Brucella blood agar plates, strips for each agent were placed onto the plates according to the manufacturer's instructions. The plates were incubated for 48 hours at 37°C in an anaerobic atmosphere. MIC was recorded at the point where the elliptical zone intersected with the strip. Since M.I.C.E. strips sometimes may lead to false results in comparison with reference agar method, all resistant strains have been retested twice. The MIC data was interpreted according to EUCAST breakpoints. In each batch a quality control for susceptibility was performed using* B. fragilis* ATCC 25285.

## 3. Results and Discussion

### 3.1. Bacterial Isolates

The 81 strains of GPAC were isolated from 72 clinical samples, obtained from the head and neck, lung, abdomen, bone and joint, and soft tissue infections of cancer patients. In nine clinical samples, two different species of GPAC were allocated at the same time. A total of 74 isolates (91,4%) were correctly identified at the species level by MALDI-TOF MS and had scores greater than 2.0. Three isolates (*Finegoldia magna*,* Peptoniphilus harei,* and* Anaerococcus vaginalis*) were identified to genus with a score between 1.8 and 2.0. Four strains (one strain of* Finegoldia magna*, two strains of* Anaerococcus vaginalis,* and one strain of* Peptoniphilus gorbachii*) could not be reliably identified and had scores below 1.7. The distribution of GPAC is shown in Table 1. The most frequent species was* Finegoldia magna*.* Peptoniphilus harei* (*Pt. harei*) was the second most frequently isolated species, followed by* Parvimonas micra* (*Pa. micra*) and* Peptostreptococcus anaerobius* (*Pe. anaerobius*), respectively. The most common sources of isolation of all GPAC were skin and soft tissue and gastrointestinal and genitourinary tracts. None of the GPAC species were isolated from blood samples during this study.

Amongst GPAC,* F. magna* is the most pathogenic organism, which was most frequently isolated in a pure culture from various clinical infection sites [1, 9]. In our study,* F. magna* was isolated in a pure culture in seven clinical samples (18,4% of all* F. magna* isolates and 8,6% of total GPAC). As a sole etiological agent,* F. magna* was isolated from patients with bone cancer after orthopedic surgery (three cases), from surgical wounds (three cases), and from a patient with cancer of the mediastinum (one case).* F. magna* was mainly isolated from surgical wounds (45%), body fluids (29%), and abscesses (24%).

*Pt. harei* was recovered from a variety of infection sources in approximately equal proportions (Table 1).* Pt. harei* frequently was isolated from clinical samples such as the body fluids (43%) and surgical wounds (39%) and rarely from abscesses (13%). All strains of* Pt. harei* were correctly identified at the species level by MALDI-TOF MS. In the past,* Pt. harei* has been often misidentified as* Pt. asaccharolyticus*, since these two species have the same biochemical characteristics and could not be differentiated from each other phenotypically [10].

*Pa. micra* and* Pe. anaerobius* were seldom isolated.* Pa. micra* was mainly isolated from infections of the lungs and abdomen, while* Pe. anaerobius* was most frequently isolated from infections of the skin and soft tissues, as well as infections of the genitourinary tract. Similar results have been obtained for* Pe. anaerobius* by other authors [11].* Pa. micra* was mainly obtained from fluids (62,5%), while* Pe. anaerobius* was most frequently isolated from surgical wounds (57%). All strains of* Pa. micra* and* Pe. anaerobius* were reliably identified at the species level by MALDI-TOF MS.

Four isolates of* Anaerococcus vaginalis* were obtained from skin and soft tissue infections, as well as wounds (*n* = 3) and subcutaneous abscess (*n* = 1). Identification of the two strains was not reliable, as species* vaginalis*/*hydrogenalis* of genus* Anaerococcus* have very similar patterns. Therefore, distinguishing their species is difficult. One strain of* Pt. gorbachii* was isolated together with* F. magna* from an ovary tumor. This was a new species of GPAC described in 2007 [12].

In a European study on antimicrobial susceptibility among 299 GPAC isolates, mainly from skin and soft tissue infections, the majority of isolates were identified as* F. magna* (37,1%),* Pa. micra* (17,7%),* Pt. harei* (14,7%),* A. vaginalis* (7,0%), and* Pe. anaerobius* (6,7%) [6]. Our data on the species diversity of GPAC is in agreement with the study, which was conducted in 10 European countries. However, there are differences in the percentage ratio of various species, in particular* Pa. micra* and* Pt. harei*, possibly owing to the more diverse sources of isolation for GPAC in our study. Furthermore, we examined the GPAC species which are encountered in cancer patients.

### 3.2. Antimicrobial Susceptibility

The MIC distributions of antimicrobial agents tested against GPAC isolates are presented in Table 2. All isolates were susceptible to imipenem, vancomycin, and linezolid; the MIC ranges were 0.004–0.12, 0.03–0.5, and 0.5–4.0, respectively. Susceptibility to penicillin G, amoxicillin/clavulanate, metronidazole, ciprofloxacin, and levofloxacin varied for different species. In our hospital, metronidazole is the drug of choice for prevention and treatment of anaerobic infections. This drug had the highest* in vitro* activity compared with imipenem and beta-lactam/beta-lactamase inhibitor combinations, in particular amoxicillin/clavulanate. For most GPAC isolates, the MIC ranges for metronidazole were 0,25–1, 0 *μ*g/mL. One* F. magna* strain isolated in pure culture from a decaying mediastinal tumor was resistant to metronidazole (32 *μ*g/mL). This strain was also resistant to penicillin G (32 *μ*g/mL), ciprofloxacin (32 *μ*g/mL), and levofloxacin (32 *μ*g/mL) while demonstrating intermediate resistance to amoxicillin/clavulanate (8 *μ*g/mL). Eight cases of postoperative mediastinitis due to* F. magna* have been described in literature, three of which were monomicrobial. However, isolated strains have been susceptible to regular antibiotics [13].

*F. magna* isolates showed the highest MIC values for penicillin (0.12 *μ*g/mL), amoxicillin/clavulanate (0.06–0.12 *μ*g/mL), and imipenem (0.03–0.06 *μ*g/mL) as compared to other GPAC. Nonetheless, all strains were susceptible to these antibiotics with the exception of one multidrug-resistant strain (Table 2). A total of 26 (68%) and 27 (71%) isolates of* F. magna* were resistant to ciprofloxacin (32 *μ*g/mL) and levofloxacin (32 *μ*g/mL), respectively. One strain with reduced susceptibility to levofloxacin (8 *μ*g/mL) was isolated. Fluoroquinolone resistance among GPAC has been reported with 14% and 27% resistance to ciprofloxacin and levofloxacin, respectively [14].

*Pt. harei* showed the highest MIC values for ciprofloxacin (1-2 *μ*g/mL) and levofloxacin (4–8 *μ*g/mL) as compared to other GPAC species. One isolate was resistant to ciprofloxacin (32 *μ*g/mL) while one strain demonstrated intermediate resistance (8 *μ*g/mL). The resistance of* Pt. harei* strains to levofloxacin was much higher than that of ciprofloxacin. A total of 12 isolates (52%) were resistant or showed intermediate resistance to levofloxacin (MIC 8–32 *μ*g/mL). With the exception of fluoroquinolones,* Pt. harei* isolates were susceptible to all tested drugs.

Among* Pa. micra*, two isolates were highly resistant to metronidazole (MIC 256 *μ*g/mL) and were sensitive to other tested antibiotics. Two isolates had reduced susceptibility to metronidazole (4 *μ*g/mL), as well as reduced susceptibility to penicillin (0,5 *μ*g/mL).* Pa. micra* strain's resistance to metronidazole has also been reported in studies from Netherlands [15]. Furthermore,* Pa. micra* strains showed the lowest MIC values for amoxicillin/clavulanate (0.015–0.03 *μ*g/mL) and imipenem (0.004–0.008 *μ*g/mL) as compared to other GPAC species (Table 2). The sensitivity of various strains of* Pa. micra* to penicillin is highly variable. Five isolates had MIC values for penicillin 0.004–0.015 *μ*g/mL, while two isolates had intermediate resistance (0.5 *μ*g/mL) and one strain was resistant to penicillin (32 *μ*g/mL). This latter strain was also resistant to levofloxacin (32 *μ*g/mL) and had reduced susceptibility to ciprofloxacin (8 *μ*g/mL). In addition, Brazier notes 8% resistance of* Pa. micra* to penicillin [16].

For* Pe. anaerobius*, penicillin showed the greatest variability. The MIC range was 0.008–32 *μ*g/mL. One strain was resistant to penicillin (1 *μ*g/mL), as well as ciprofloxacin and levofloxacin (32 *μ*g/mL for each). The second strain was only resistant to penicillin (32 *μ*g/mL). All* Pe. anaerobius* isolates were susceptible to metronidazole, amoxicillin/clavulanate, and imipenem. However, three strains were resistant to ciprofloxacin and levofloxacin (32 *μ*g/mL). In contrast to our data, 10% of* Pe. anaerobius* isolates showed resistance to amoxicillin/clavulanate [11]. The combination of amoxicillin/clavulanic acid has been reported as being less effective against* Pe. anaerobius* isolates as compared to other GPAC [16].

All* A. vaginalis* isolates were resistant to ciprofloxacin and levofloxacin (32 *μ*g/mL) but were sensitive to other tested antibiotics. One* Pt. gorbachii* isolate was susceptible to all drugs, with the exception of levofloxacin (16 *μ*g/mL). The resistance of* A. vaginalis* and* Pt. gorbachii* isolates to levofloxacin was noted by other authors [12, 15].

In conclusion, correct identification of GPAC to species level and their susceptibility to antimicrobial agents is necessary in order to administer effective antibiotic therapy, as such data can vary substantially in different clinics. Furthermore, much consideration must be given to the reduced susceptibility of GPAC to penicillin, as well as frequent reports of resistance to metronidazole, which are often used in empirical antimicrobial therapy of infections caused by GPAC.

## Supplementary Material

The 81 gram-positive anaerobic cocci were isolated from cancer patients. The majority of isolates were identified as *Finegoldia magna* (47%) and *Peptoniphilus harei* (28%) by MALDI-TOF MS. One *Finegoldia magna* isolate was resistant to metronidazole (32 *µ*g/mL), penicillin G (32 *µ*g/mL), ciprofloxacin, and levofloxacin (32 *µ*g/mL) while demonstrating intermediate resistance to amoxicillin/clavulanate (8 *µ*g/mL). Two Pa. micra isolates were highly resistant to metronidazole (256 *µ*g/mL) and were sensitive to other tested antibiotics. All isolates were susceptible to imipenem, vancomycin, and linezolid. Susceptibility to penicillin G, amoxicillin/clavulanate, metronidazole, ciprofloxacin, and levofloxacin varied for different species.

## Figures and Tables

**Table 1:** Distribution and source of isolation of different species of GPAC between 2004 and 2014.

Species	Number of strains isolated from infections of the following						
	Head and neck	Lung	Abdomen	Skin and soft tissue	Bone and joint	Genitourinary system	Total (%)
*Finegoldia magna*	2	2	9	12	5	8	38 (46,9)
*Peptoniphilus harei*	2	4	6	4	2	5	23 (28,4)
*Parvimonas micra*		3	4		1		8 (9,9)
*Peptostreptococcus anaerobius*	1			3		3	7 (8,6)
*Anaerococcus vaginalis*				4			4 (4,9)
*Peptoniphilus gorbachii*						1	1 (1,2)
Total	5	9	19	22	8	18	81

**Table 2:** MIC distribution of antimicrobial agents tested against GPAC isolates.

Organism (number of strains)	Antimicrobial agent	Number of isolates for which the antimicrobial agent MIC (*μ*g/mL) was as follows													
		0.004	0.008	0.015	0.03	0.06	0.12	0.25	0.5	1	2	4	8	16	≥32
*F. magna* (38)	Penicillin G			1		7	24	5							1
	Amoxicillin/clavulanate			1	1	14	13	7	1				1		
	Imipenem			2	16	17	2				1				
	Metronidazole				1	1	2	6	12	13	2				1
	Ciprofloxacin					1	2	3	5		1				26
	Levofloxacin						1	1	4	4			1		27

*Pt. harei* (23)	Penicillin G		1	2	3	11	5								
	Amoxicillin/clavulanate		1	5	7	7	1		1						
	Imipenem	1	6	8	4	1	2								
	Metronidazole						3	5	6	7	1				
	Ciprofloxacin								1	7	11	1	1		1
	Levofloxacin										1	9	8	1	3

*Pa. micra* (8)	Penicillin G	2	2	1					2						1
	Amoxicillin/clavulanate			6	2										
	Imipenem	2	5	1											
	Metronidazole							1	2	1		2			
	Ciprofloxacin							5	1	1			1		
	Levofloxacin							5	1	1					1

*Pe. anaerobius* (7)	Penicillin G		1		1	1	1	1		1					1
	Amoxicillin/clavulanate				1	2	4								
	Imipenem			3	4										
	Metronidazole					1	1	2	3						
	Ciprofloxacin								1	3					3
	Levofloxacin							1	3						3

*A. vaginalis* (4)	Penicillin G				2	2									
	Amoxicillin/clavulanate			1	2	1									
	Imipenem			3	1										
	Metronidazole							4							
	Ciprofloxacin														4
	Levofloxacin														4

*Pt. gorbachii* (1)	Penicillin G					1									
	Amoxicillin/clavulanate			1											
	Imipenem		1												
	Metronidazole							1							
	Ciprofloxacin										1				
	Levofloxacin													1	
