# Supplementary material for: Evaluation of Antibiotic Susceptibility of Gram-Positive Anaerobic Cocci Isolated from Cancer Patients of the N. N. Blokhin Russian Cancer Research Center
Source: J Pathog. 2015 Dec 21;2015:648134. doi: 10.1155/2015/648134 (PMC4698780; doi:10.1155/2015/648134)
Supplement: Supplementary file 1 — The 81 gram-positive anaerobic cocci were isolated from cancer patients. The majority of isolates were identified as Finegoldia magna (47%) and Peptoniphilus harei (28%) by MALDI-TOF MS. One Finegoldia magna isolate was resistant to metronidazole (32 µg/mL), penicillin G (32 µg/mL), ciprofloxacin, and levofloxacin (32 µg/mL) while demonstrating intermediate resistance to amoxicillin/clavulanate (8 µg/mL). Two Pa. micra isolates were highly resistant to metronidazole (256 µg/mL) and were sensitive to other tested antibiotics. All isolates were susceptible to imipenem, vancomycin, and linezolid. Susceptibility to penicillin G, amoxicillin/clavulanate, metronidazole, ciprofloxacin, and levofloxacin varied for different species. [file 648134.f1.docx]

- The 81 gram-positive anaerobic cocci were isolated from cancer patients.
- The majority of isolates were identified as *Finegoldia magna* (47%) and *Peptoniphilus harei* (28%) by MALDI-TOF MS.
- One *Finegoldia magna* isolate was resistant to metronidazole (32 µg/ml), penicillin G (32 µg/ml), ciprofloxacin, and levofloxacin (32 µg/ml) while demonstrating intermediate resistance to amoxicillin/clavulanate (8 µg/ml).
- Two *Pa. micra* isolates were highly resistant to metronidazole (256 µg/ml) and were sensitive to other tested antibiotics.
- All isolates were susceptible to imipenem, vancomycin, and linezolid. Susceptibility to penicillin G, amoxicillin/clavulanate, metronidazole, ciprofloxacin, and levofloxacin varied for different species.
